# Printable Carbon Black/Graphite Conductive Ink toward Electrochemical Determination of Norfloxacin in Environmental Samples

**DOI:** 10.1021/acsomega.5c08667

**Published:** 2025-10-10

**Authors:** Marcella Matos Cordeiro Borges, Thaís Cristina de Oliveira Cândido, Arnaldo César Pereira

**Affiliations:** Departamento de Ciências Naturais, Universidade Federal de São João del-Rei (UFSJ), Campus Dom Bosco, Praça Dom Helvécio 74, Fábricas, 36301-160 São João del-Rei, Minas Gerais, Brazil

## Abstract

Norfloxacin (NOR), a fluoroquinolone antibiotic widely used to treat urinary tract infections and gastrointestinal disorders caused by bacteria, poses a significant environmental concern. The widespread use of NOR can lead to the development of antibiotic resistance in human populations and, upon release into the environment, can contaminate aquatic and terrestrial ecosystems, potentially impacting human and environmental health. This work aimed to develop an electrochemical sensor printed with a conductive ink composed of graphite (Gr), carbon black (CB), and stained-glass varnish (SGV) for the detection of NOR in environmental samples. The optimal ink composition demonstrated 33% Gr, 22% CB, and 45% SGV. The morphology of the proposed sensor was designed by scanning electron microscopy and Fourier transform infrared spectroscopy, and electrochemical characterization was performed by cyclic voltammetry and electrochemical impedance spectroscopy. Differential pulse voltammetry was employed for NOR determination using 0.05 mol L^–1^ Britton-Robinson buffer as the electrolyte. Optimal current responses were obtained at pH 5.0. The developed sensor presented a detection limit of 0.227 μmol L^–1^ and a quantification limit of 0.756 μmol L^–1^ for NOR, demonstrating high sensitivity, precision, and accuracy. Furthermore, its application in real river water samples provided recovery values between 83.9 and 103.3%. The sensor proved suitable and stable for NOR determination in environmental samples.

## Graphical abstract

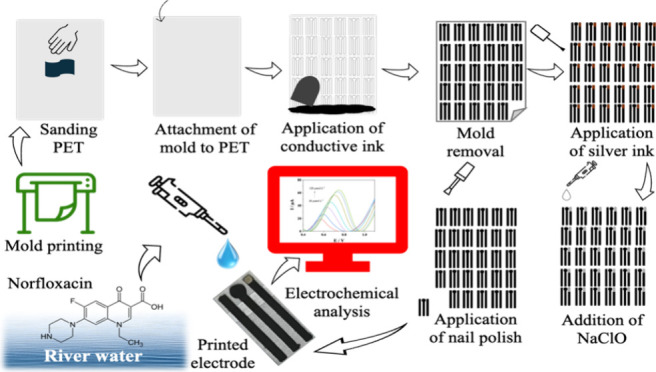

## 1 Introduction

Many pharmaceutical products for human or veterinary use, as well as pesticides, are used throughout the world. Aquatic environments have been suffering increasing contamination by pharmaceutical compounds called emerging pollutants, that is, synthetic or natural chemical substances that are not monitored and even in low concentrations can cause risks to human health and the environment.^1–3^ The excessive and indiscriminate use of antimicrobial drugs used in the treatment of infectious diseases caused by bacteria has become a public health problem, since new mechanisms of resistance to these drugs are emerging and spreading in all regions of the world.^4,5^

Antibacterial agents of the quinolone or 4-quinolone class are composed of diverse groups, with their actions based on anti-DNA activities. Antibiotics belonging to the quinolone group have a carboxylic group in position 3 and a carbonyl group in position 4.^6^ Fluoroquinolones were created by replacing a fluorine moiety at position 6.^7^ Although nonfluorinated quinolones have only a moderately extended Gram-negative spectrum, fluoroquinolones were developed for activity against Gram-negative and Gram-positive organisms, chlamydiae, and mycoplasmas. Fluoroquinolones include norfloxacin (NOR), enrofloxacin, ciprofloxacin, orbifloxacin, lomefloxacin, ofloxacin, danofloxacin, flumequine, difloxacin, and marbofloxacin, among others. The mechanism of action of fluoroquinolones is interference with nucleic acid synthesis.^6,7^

NOR was the first member of the second-generation fluoroquinolone group to present a spectrum that covers both Gram-positive and Gram-negative bacteria (including *Pseudomonas aeruginosa*). In humans, NOR is considered a very effective antibiotic for urinary tract infections, but not for systemic infections. This drug has been used in dogs for Enterococcus infections, as it presents better results compared to ciprofloxacin or enrofloxacin.^8^ The importance of removing this drug from the environment is mainly due to its ability to promote bacterial resistance,^9,10^ in addition to interfering with several systems in the environment.

Given the need for fast and accurate analytical methods for monitoring these types of antibiotics in environmental matrices, screen-printed electrodes emerge as a promising alternative due to their portability and low-cost characteristics.^11^ The screen-printing technique not only facilitates the miniaturization and mass production of electrochemical sensors at significantly reduced costs but also allows the use of various nonconductive substrates, such as glass, plastic, or ceramic material, and conductive inks composed of different conductive materials, such as graphite, carbon black, or graphene, allowing the optimization of sensor performance for different applications.^12^

Fernandez et al. employed a screen-printed graphene electrode for paracetamol detection, emphasizing its large surface area and strong electrocatalytic properties. This electrode exhibited excellent performance in paracetamol detection, featuring a low detection limit of 20 nmol L^–1^ and high selectivity even in complex biological samples such as human oral fluids without the need for sample pretreatment or electrode modification. This simplicity and cost-effectiveness make it a promising candidate for real-time applications.^13^ Fabiani and collaborators developed the first sensitive electrochemical biosensor for detecting SARS-CoV-2 in saliva using a screen-printed carbon black electrode as the sensor, combined with a portable potentiostat as the reader. A detection limit of 19 and 8 ng mL^–1^ was obtained in untreated saliva, respectively, for protein S and N. The low detection limit achieved, the rapid analysis (30 min), the miniaturization and portability of the instrument, combined with the ease of use and noninvasive sampling, give this analytical tool high potential for market entry as the first highly sensitive electrochemical immunoassay for detecting SARS-CoV-2 in untreated saliva.^14^

In view of the growing need for portable and low-cost analytical methods for environmental monitoring, this study proposed the development of printable conductive ink composites of carbon black (CB) and graphite (Gr) toward the electrochemical determination of NOR in environmental samples. This device was obtained by screen printing on a polyethylene terephthalate (PET) substrate, using ink composed of carbon black/graphite, bonded with stained-glass varnish, and dispersed in acetone. The resulting electrode has been characterized by scanning electron microscopy (SEM), Fourier transform infrared spectroscopy (FTIR), and electrochemical techniques. Finally, the analytical method was applied to the determination of NOR in river water samples.

## 2 Experimental Section

### 2.1 Solvents, Reagents, Standard and Stock Solutions

Pure powdered Gr, acetone, and phosphoric acid were purchased from Synth (Diadema, SP, Brazil). NOR, azithromycin, prednisolone, acetic acid, boric acid, and methanol were obtained from Sigma-Aldrich (St. Louis, MO, USA). Lomefloxacin was purchased from Chem-Gold (DaJOTA, SP, Brazil). Potassium ferricyanide (K_3_[Fe­(CN)_6_]) and potassium ferrocyanide (K_4_[Fe­(CN)_6_]) were obtained from Neon (Suzano, SP, Brazil). CB powder was purchased from Cabot (Maua, SP, Brazil). All chemical reagents used were of analytical grade. Stained-glass varnish was purchased from Acrilex (São Bernardo do Campo, SP, Brazil). Stock solutions of NOR, azithromycin, prednisolone, and lomefloxacin were prepared in methanol. All other solutions were prepared in water purified by a Millipore (Burlington, MA, USA) Milli-Q system.

### 2.2 Printable Conductive Ink Preparation

The printable conductive ink was prepared by mixing 1.8 g of Gr, 0.2 g of CB, and 2.0 g of SGV. To completely homogenize the ink, 5 mL of acetone was added. The Gr, CB, and SGV ratios were optimized. Figure 1A illustrates the detailed procedure for the ink preparation. A comparative study of ink compositions was conducted, including a Gr-based ink (2.0 g Gr, 2.0 g SGV, 5.0 mL acetone).

**Figure 1:**
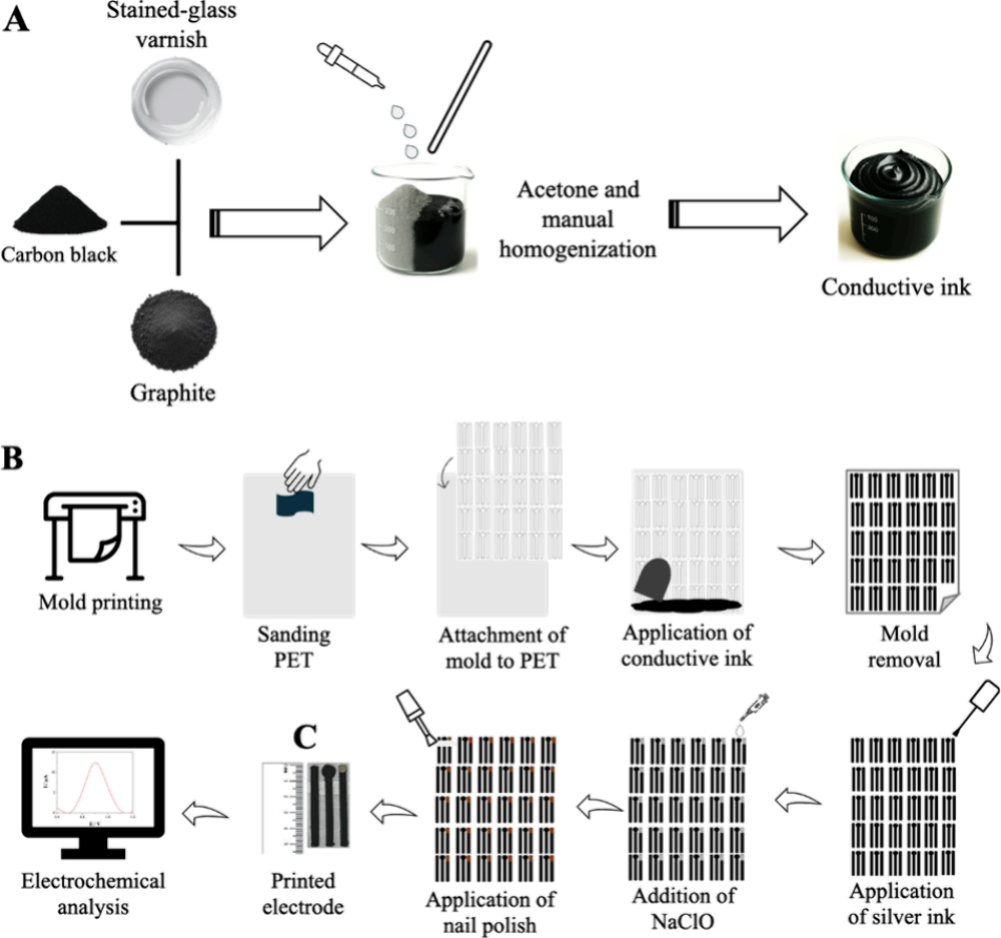
Illustrative scheme of (A) printable conductive ink preparation using graphite, carbon black, stained-glass varnish, and acetone; (B) of the process of obtaining the printed electrode; and (C) finished printed electrode.

### 2.3 Printed Electrode Fabrication and Development

The silk-screen printing technique was employed to fabricate disposable printed electrodes by using the prepared ink, which incorporated the auxiliary, working, and quasi-reference electrodes. Initially, the molds were designed using CorelDraw X6 software and printed on adhesive vinyl sheets with a Visutec MVSK800 cutting plotter. These molds were then affixed to a PET sheet that had been previously sanded and cleaned with 70% ethyl alcohol, serving as the substrate. Subsequently, the conductive ink was uniformly deposited over the molds by using a squeegee to ensure uniform distribution. After drying, the molds were removed, and the electrodes were carefully cut out. The construction of the quasi-reference electrode involved the deposition of silver ink,^15^ followed by the addition of a sodium hypochlorite aliquot. This step promoted the formation of silver chloride (AgCl), resulting in a Ag/AgCl electrode. The final step of the process involved applying a layer of nail polish to define the geometric area of the electrodes and insulate the electrical contact region from measuring equipment. Figure 1B illustrates the electrode fabrication process.

### 2.4 Instrumentation

Morphological characterization of the CB, Gr, SGV, and conductive ink samples was carried out using SEM with a JEOL JSM-300LV microscope. FTIR analysis was conducted on the same samples with a Shimadzu PerkinElmer Spectrum GX Series FTIR-8300 spectrometer. Spectra were recorded using KBr pellets in the range of 4000 to 500 cm^–1^. Electrochemical studies were conducted by using cyclic voltammetry (CV), electrochemical impedance spectroscopy (EIS), and differential pulse voltammetry (DPV). Measurements were carried out with an Autolab PGSTAT204 potentiostat/galvanostat operated via Nova 2.1 software for data acquisition. The resulting data were analyzed using OriginPro 2018 software.

## 3 Results and Discussion

### 3.1 Electrochemical Characterization

The electrode developed on a PET substrate offers the advantages of flexibility and miniaturization, making it suitable for electrochemical analyses (Figure 1C). The proposed electrode has been compared with a printed electrode configuration using ink containing only Gr and SGV. The carbonaceous materials (Gr and CB) provided electrical conductivity to the ink SGV, a low-cost and widely available binder, which was used to promote cohesion among the conductive particles. PET was chosen as the substrate because of its chemical inertness, affordability, and excellent mechanical and thermal properties.^16^

The electrochemical performance of the printed material can be influenced by the proportions of the ink constituents, which were optimized using the DPV. Figure 2A illustrates the characteristic voltammograms of each ink formulation. The measurements were performed in the presence of 0.2 mmol of L^–^^1^ NOR and 0.1 mol of L^–^^1^ Britton–Robinson buffer at pH 7.0. The amount of SGV was fixed at 2.0 g. Among the various compositions evaluated, the ink with a mass ratio of 40% CB and 60% Gr demonstrated superior electrochemical performance, as evidenced by a higher analytical signal for NOR and good adhesion to the PET substrate. The ink formulation with a 50:50 Gr/CB ratio showed a decrease in current and, consequently, inferior electrochemical performance. Moreover, this formulation exhibited poor adhesion to the PET substrate, which negatively affected the electrode’s stability. This issue may be attributed to the larger volume occupied by CB compared to Gr.^17^ Ink compositions with a reduced proportion of CB exhibited an inferior electrochemical performance, characterized by less defined peaks. This behavior may be attributed to the insufficient amount of CB, given its essential role in enhancing conductivity and the electroactive surface area. Therefore, the 60% Gr and 40% CB formulation was selected for optimizing the SGV content.

**Figure 2:**
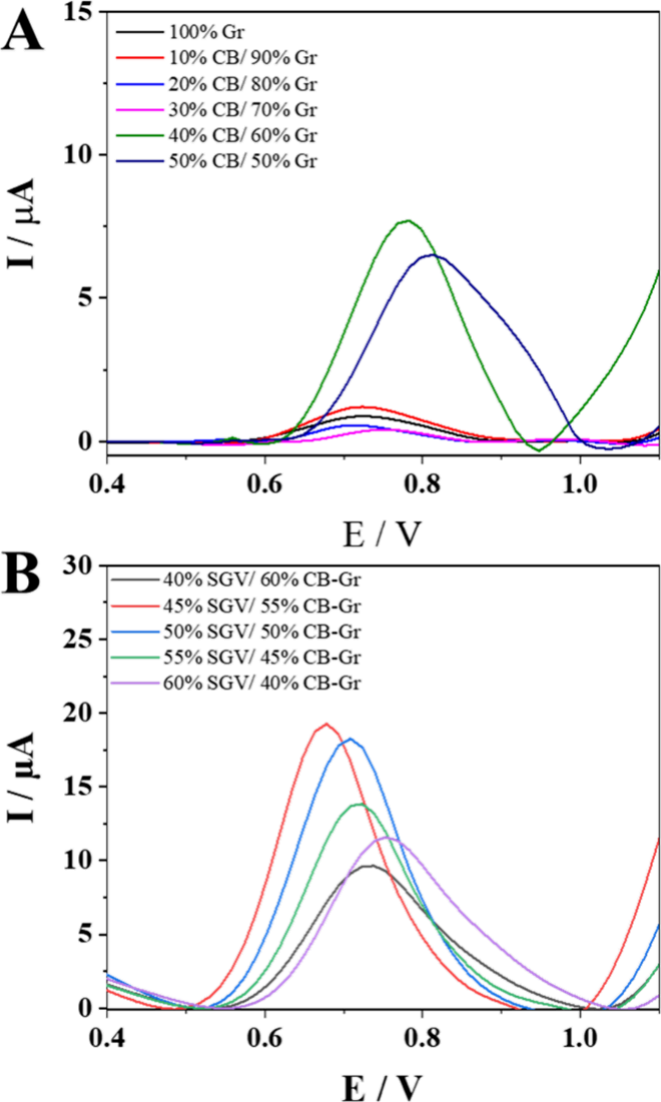
Differential pulse voltammograms obtained for (A) different Gr and CB compositions and (B) different SGV ratios. Experimental conditions: 0.20 mmol L^–1^ NOR in 0.1 mol L^–1^ Britton–Robinson buffer, pH 7.0.

For the evaluation of the SGV proportion, a fixed amount of 2.0 g of carbonaceous material was used. Figure 2B shows that the ink containing 40% SGV was less suitable for application, exhibiting a lower current response and poor adhesion to the PET substrate. When the SGV percentage was varied from 45 to 50%, no significant changes were observed in the voltametric profile. Conversely, inks formulated with 55 and 60% binder showed a decrease in current intensity. This suggests that the increased electrical resistance may be attributed to the excessive proportion of the insulating binder in the ink composition, thereby reducing the electrode’s efficiency. Due to its good homogeneity and superior voltametric profile, the ink containing 45% SGV was selected for the subsequent phases of the study.

#### 3.1.1 Kinetic Study and Determination of the Sensor’s Electroactive Area

The electroactive surface area of the printed electrode was determined by CV in a 5.0 mmol L^–1^ K_4_[Fe­(CN)_6_] solution prepared in 0.1 mol L^–1^ of phosphate buffer at pH 7.0. The results were compared to those of a printed electrode fabricated with ink containing only Gr and SGV. The cyclic voltammograms obtained are presented in Figure S1. The results demonstrate a direct correlation between the scan rate and current intensity, where an increase in scan rate results in a corresponding increase in peak current. The linear correlation observed between the anodic and cathodic peak currents and the square root of the scan rate, as described by the Randles–Ševčík eq 1,^18^ is characteristic of reversible processes in which mass transport is governed by diffusion, in which the diffusion coefficient was 7.6 × 10^–6^ cm^2^ s^–1^.

$$i_{p}=2686\times 10^{5}\times n^{3/2}\times A\times C\times (D\cdot \nu {)}^{1/2}$$ 1

where *i*_p_, anodic or cathodic peak current (Ampere); *n*, number of electrons involved in the redox reaction (dimensionless constant) – *n* = 1; *A*, electroactive area of the electrode (cm^2^); *D*, diffusion coefficient (cm^2^ s^–1^) – *D* = 7.6 × 10^–6^ cm^2^ s^–1^; *C*, concentration of reduced or oxidized species (mol cm^3^) – *C* = 5 × 10^–6^ mol cm^3^; ν, scan speed (mV s^–1^).

The calculations performed indicated an electroactive area of 5.0 mm^2^ for the printed electrode using Gr ink and 11.0 mm^2^ for the electrode using Gr and CB ink. The difference in the electroactive area between the electrodes can be attributed to the ink composition. The incorporation of CB enhanced the sensor′s performance due to its high surface area and excellent conductive properties. A comparison between the electrodes was carried out, and the results showed that the Gr–CB electrode exhibits higher oxidation currents compared to the graphite-only electrode (Figure S2). This increase is consistent with an enlargement of the electrochemically active surface area, demonstrating that the incorporation of CB enhances the electrocatalytic performance of the electrode.

#### 3.1.2 Electrochemical Impedance Spectroscopy (EIS)

EIS was employed to investigate the electrical properties of the electrode/solution interface, allowing for the characterization of charge transfer processes and electric double layer behavior, as influenced by the individual ink constituents. The experiments were carried out in 0.1 mol L^–1^ KCl containing 5.0 mmol L^–1^ K_3_[Fe­(CN)_6_]/K_4_[Fe­(CN)_6_], applying the open-circuit potential (OCP) with an amplitude of 0.1 V over a frequency range from 100 kHz to 0.1 Hz for both Gr-SGV/PET and Gr-CB-SGV/PET electrodes.

The Nyquist plot and equivalent circuit are shown in Figure S3A. The charge transfer resistance (*R*_ct_) for the Gr-SGV/PET sensor was 5.7 kΩ, whereas the *R*_ct_ for the Gr-CB-SGV/PET sensor was 2.44 kΩ. The higher *R*_ct_ observed for the ink containing only Gr indicates that Gr-CB-SGV/PET offers lower impedance to electron transfer at the electrode–electrolyte interface. These results demonstrate that the ink containing both Gr and CB exhibits superior conductivity and more favorable electrochemical kinetics, likely due to the large surface area and conductive nature of CB.^19^

#### 3.1.3 Cycle Stability of the Sensor

The stability of the developed printed sensor containing both Gr and CB has also been investigated using cyclic voltammetry over 50 consecutive cycles. Measurements were performed at a scan rate of 30 mV s^–1^ in a 0.1 mol L^–1^ phosphate buffer solution at pH 7.0 containing 5.0 mmol L^–1^ K_4_[Fe­(CN)_6_]. The cyclic voltammograms obtained are shown in Figure S3B. The sensor exhibited only a 7.7% decrease in the intensity of the signal after 50 cycles, as observed from the anodic peak response, indicating high stability. These findings suggest that the printed electrodes fabricated with the developed ink have a strong potential for use in analytical applications. The slight signal loss did not compromise the sensor’s long-term performance, implying good adhesion of the ink to the PET substrate.

### 3.2 Materials Characterization

#### 3.2.1 SEM

SEM was employed to study the morphology of the precursor materials (SGV, Gr, and CB) and the conductive ink. The resulting images, captured at 1000× magnification, are presented in Figure 3. The SEM image of SGV (Figure 3A) reveals a continuous and uniform film, which supports the material’s resinous characteristics.^20^ The micrograph of Gr (Figure 3B) highlights its characteristic lamellar structure, with irregular individual flakes.^21^ For CB (Figure 3C), it is possible to observe a heterogeneous morphology with aggregates of spherical particles of different sizes. Generally, these agglomerates have diameters ranging from 1 to 100 μm and are held together by van der Waals forces.^22^ The SEM image of the conductive ink (Figure 3D) reveals the formation of a granular structure, indicating a large surface area, which is desirable for conductive ink applications.

**Figure 3:**
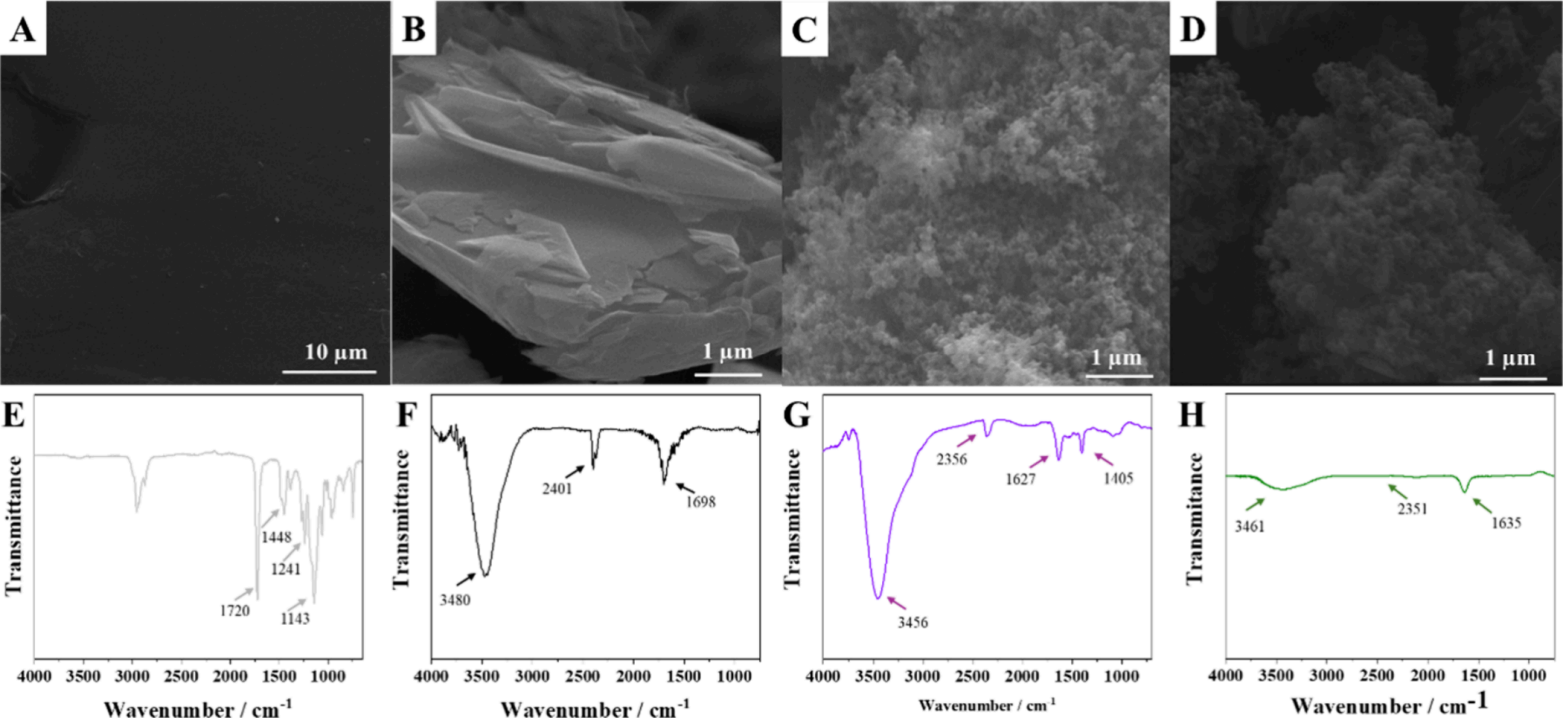
Electron micrographs at a magnification of 1000× of (A) SGV, (B) Gr, (C) CB, and (D) conductive ink (Gr-CB-SGV); FTIR spectra for (E) SGV, (F) Gr, (G) CB, and (H) conductive ink (Gr-CB-SGV).

#### 3.2.2 FT-IR

Figure 3 shows FT-IR spectra for SGV, Gr, CB, and the conductive ink. The FTIR spectrum of SGV (Figure 3E) presents bands characteristic of alkyd resins, such as the asymmetric (1720 cm^–1^) and symmetric (1448 cm^–1^) vibration of the carbonyl group (C=O) of esters, confirming the presence of this functional group in the resin structure. The bands observed at 1241 and 1143 cm^–1^ are attributed to the stretching vibrations of the C–O–C and C–O bond.^23,24^ In the FTIR spectrum for Gr (Figure 3F), there is a band at 3480 cm^–1^ corresponding to the −OH group, which may be due to the adsorption of water molecules on the surface of the material. The band observed at 1698 cm^–^^1^ is attributed to the stretching vibration of the C=C bond, characteristic of carbonaceous materials.^25^ The band around 2401 cm^–1^ observed in the spectra of Gr and CB at 2356 cm^–1^ and the ink at 2351 cm^–1^ indicates the possible adsorption of atmospheric carbon dioxide on the surface of these materials.^26^ The FTIR spectrum of CB shows the presence of a characteristic band at 3456 cm^–1^ of −OH groups (Figure 3G). The signal at 1627 cm^–1^ is attributed to the stretching vibration of the C=C bond, and the presence of a band at 1405 cm^–1^ is characteristic of the carbonyl stretching vibration.^27^ The FTIR characterization of CB confirmed the presence of oxygen-containing functional groups on its surface, which may influence its adsorption properties and interactions with other molecules.

The band at 1635 cm^–1^ in the FTIR spectrum of the conductive ink is attributed to the stretching vibration of the C=C bond, characteristic of carbonaceous materials, thus confirming the presence of Gr and CB in the conductive ink composition (Figure 3H). The spectrum shows the band at 3461 cm^–1^ attributed to hydroxyl groups due to the presence of water.^26^ The nonidentification of the characteristic bands of SGV in the FTIR spectrum of the conductive ink may be related to the union and the high amount of Gr and CB exposed on the electrode surface, partially blocking the exposure of SGV.^25^

### 3.3 Electrochemical Performance: A Study of pH and Buffer Solution Concentration

The effect of pH was studied in the range of 3.0 to 11.0 using 0.1 mol L^–1^ Britton–Robinson buffer to evaluate the influence of the predominant chemical species of the analyte on the electrochemical response. NOR has two functional groups in its structure responsible for its ionization in different pH ranges: the acidic 3-carboxylic group and the basic nitrogen of the piperazine ring. Thus, the NOR ionization profile is characterized by three ionic species: the cationic form, predominant at acidic pH (below 6.22), the anionic form, predominant at alkaline pH (above 8.51), and the zwitterionic form (electrically neutral), which coexists in an intermediate pH range.^28^ Thus, the electrochemical response of NOR can be influenced by the pH of the medium, as shown in Figure 4. The peak current intensity exhibited a gradual increase with the pH, reaching a maximum at pH 5.0. At higher pHs (pH 7.0, 9.0, and 11.0), there was a significant decrease in current, indicating a lower efficiency in electron transfer and, consequently, in analyte detection. Therefore, pH 5 was chosen for the determination of NOR.

**Figure 4:**
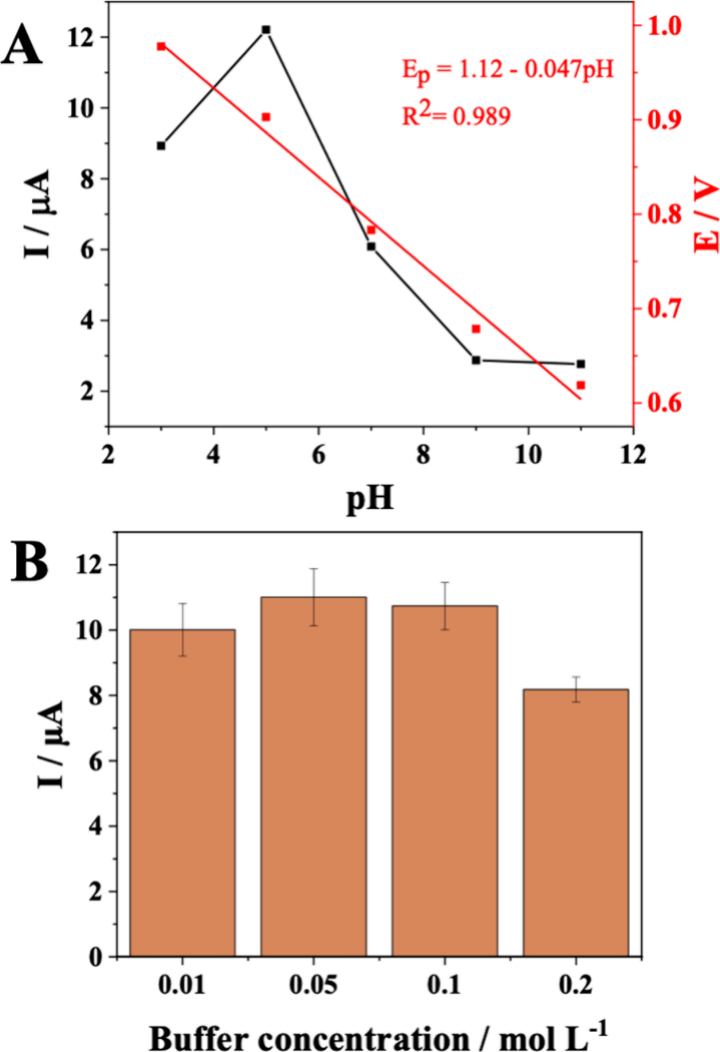
(A) Variation of cathodic peak current and potential with buffer solution pH. Reaction medium: 0.20 mmol L^–1^ of NOR in 0.1 mol L^–1^ Britton–Robinson buffer; (B) Effect of ionic strength of Britton–Robinson buffer on the electrochemical response of NOR.

The anodic peak current and potential values of the NOR demonstrated a significant dependence on the pH of the solution, which indicates its influence on the oxidation kinetics of the NOR. Figure 4A shows that the peak current intensity (*I*_p_) increases at pH 5.0, followed by decay and subsequent stabilization at pH values above 9.0. At the same time, the peak potential (*E*_p_) exhibited a shift to lower values with an increase in pH, which suggests the participation of protons in the electrochemical process. The dependence of *E*_p_ on pH can be described by the Nernst equation (eq 2), where *E*_p_ is the peak potential, *m* and *n* are the numbers of electrons and protons participating in the reaction, and *b* is the linear coefficient.^29^

$$E_{p}(V)=-0.059(m/n)\mathrm{pH}+b$$ 2

The experimental value of the peak potential change per pH unit (−0.047) is in good agreement with the theoretical value predicted by the Nernst equation, suggesting that the NOR oxidation process involves an equal number of protons and electrons. Initially, NOR adsorbs onto the surface of the modified electrode, interacting with the active sites through π–π interactions, hydrophobic, and chemical forces. During oxidation, the piperazine group loses two electrons and two protons, forming oxidized intermediates, such as cationic radicals or imino groups. Simultaneously, the quinolone ring undergoes partial polarization, which facilitates electron transfer. As a result, NOR is converted into oxidized products, explaining the high sensitivity observed in its electrochemical detection.^30^

The influence of ionic strength on the sensor response was investigated by varying the concentration of the Britton-Robinson buffer at pH 5.0, since ionic mobility in the solution can influence the charge transfer process at the electrode/solution interface. Concentrations of 0.01, 0.05, 0.1, and 0.2 mol L^–1^ were evaluated. Analysis of the data presented in Figure 4B shows that no significant variations in signal intensity were observed for concentrations of 0.01, 0.05, and 0.1 mol L^–1^. However, very low concentrations, such as 0.01 mol L^–1^, can compromise the stability of the ionic strength of the solution, reducing the efficiency of the charge transfer. On the other hand, the significant decrease in current observed at 0.2 mol L^–1^ can be attributed to interactions between the electrolyte ions and the NOR molecules, resulting in a possible masking of the analytical signal. Therefore, considering that the concentration of 0.05 mol L^–1^ provided the highest signal intensity, this was established as the best condition for the determination of the NOR, ensuring high sensitivity to the method.

### 3.4 Optimization of the Sensor Design and Operating Parameters of the VPD

Different printed electrode designs were evaluated, aiming to identify the configuration that presents the best electrochemical performance. Three electrode designs (Figure 5A) were subjected to electrochemical tests in the presence of the analyte of interest (NOR). The anodic peak current intensity was used as a parameter to evaluate the performance of the different designs. The analysis of the data presented in Figure 5B demonstrates that Design 3 presented the highest peak current intensity, evidencing superior electrochemical performance in relation to the others. The circular geometry of working electrodes 2 and 3 favored mass transfer and improved the kinetics of the electrochemical reactions, resulting in higher current intensities. Based on these results, design 3 was selected for the subsequent experimental stages.

**Figure 5:**
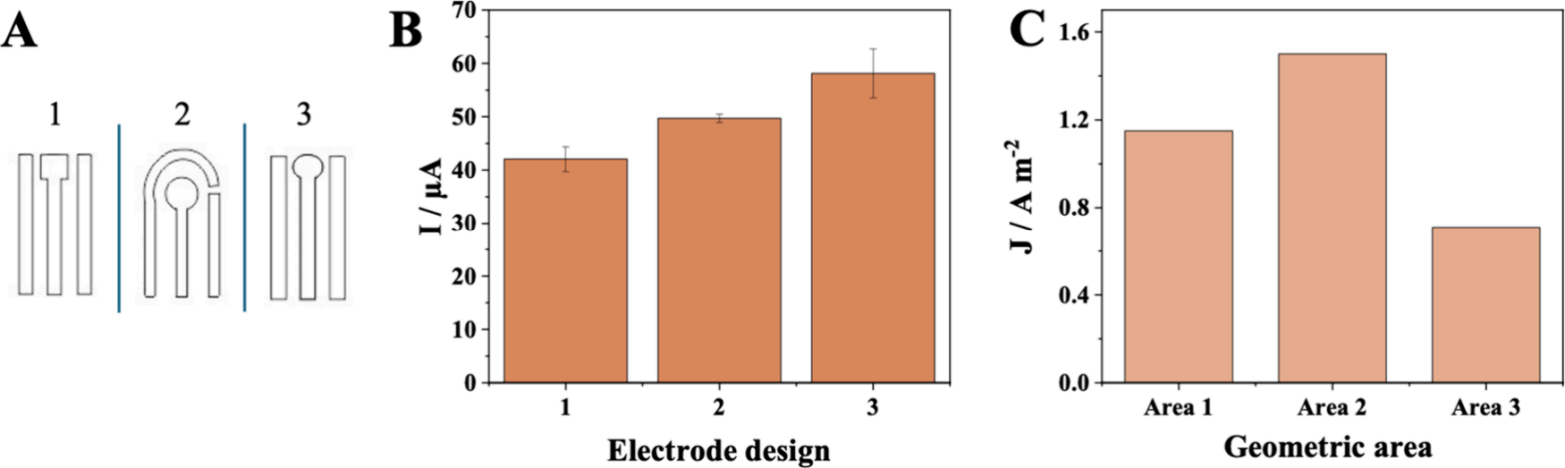
(A) Different electrode designs; (B) Variation of peak current intensity as a function of the electrode design; (C) Current density – *J* – for each area of the working electrode. Reaction medium: 0.20 mmol L^–1^ NOR in 0.05 mol L^–1^ Britton-Robinson buffer at pH 5.

In addition to the design, three sizes of the geometric area of the working electrode were also studied: *A*_1_ = 18.40 mm^2^, *A*_2_ = 28.74 mm^2^, and *A*_3_ = 32.78 mm^2^. The current density was determined by dividing the measured current by the geometric area of the electrode, allowing for a more accurate comparison of the performance of the different sensors. The electrode that presented the best electrochemical performance was the device with Area 2 (*A*_2_), as shown in Figure 5C. At a smaller size, there was a lower current gain, and at a larger size (*A*_3_), the intensity decreased by about 1 × *A*_2_. Thus, the size of the working electrode area selected as the best measurement for printing the electrodes was 28.74 mm^2^.

DPV was the main technique used in this work to minimize the contribution of the capacitive current, increasing the sensitivity of the measurement and allowing a better characterization of the electrochemical process. The optimized settings for the DPV were amplitude (0.005–0.4 V) and velocity (0.005–0.03 V s^–1^), defined in Britton–Robinson 0.1 mol L^–1^ at pH 5 and the presence of NOR 0.2 mmol L^–1^. The optimal conditions were 0.3 V for amplitude and 0.02 V s^–1^ for velocity.

### 3.5 Figures of Merit

After optimization of the experimental conditions, the analytical applicability of the printed electrode was evaluated by constructing an analytical curve. The determinations were performed using the DPV in Britton-Robinson buffer 0.05 mol L^–1^ and pH 5.0. Figure 6A shows the voltammograms obtained, in which a gradual increase in the peak current is observed with an increase in the NOR concentration. The corresponding analytical curve (Figure 6B), constructed from the relationship between the peak current and the NOR concentration, exhibited excellent linearity, according to the equation *I*_pa_ (μA) = 13.627 + 0.43 [NOR], in the range of 20 to 120 μmol L^–1^. The limit of detection (LOD) and limit of quantification (LOQ) were determined to be 0.227 and 0.756 μmol L^–1^, respectively. The precision (relative standard deviation – RSD%) of the method was assessed by means of repeatability and intermediate precision using the concentrations 20, 70, and 120 μmol L^–1^, with three replicates for each point were 13.4, 1.6, and 7.2%, respectively. Accuracy that determined by means relative error percentage (RE%) using the same points, presented −10.3, 4.0, and −10.5%, respectively. The data obtained demonstrates the potential of using the printed sensor in electrochemical applications.

**Figure 6:**
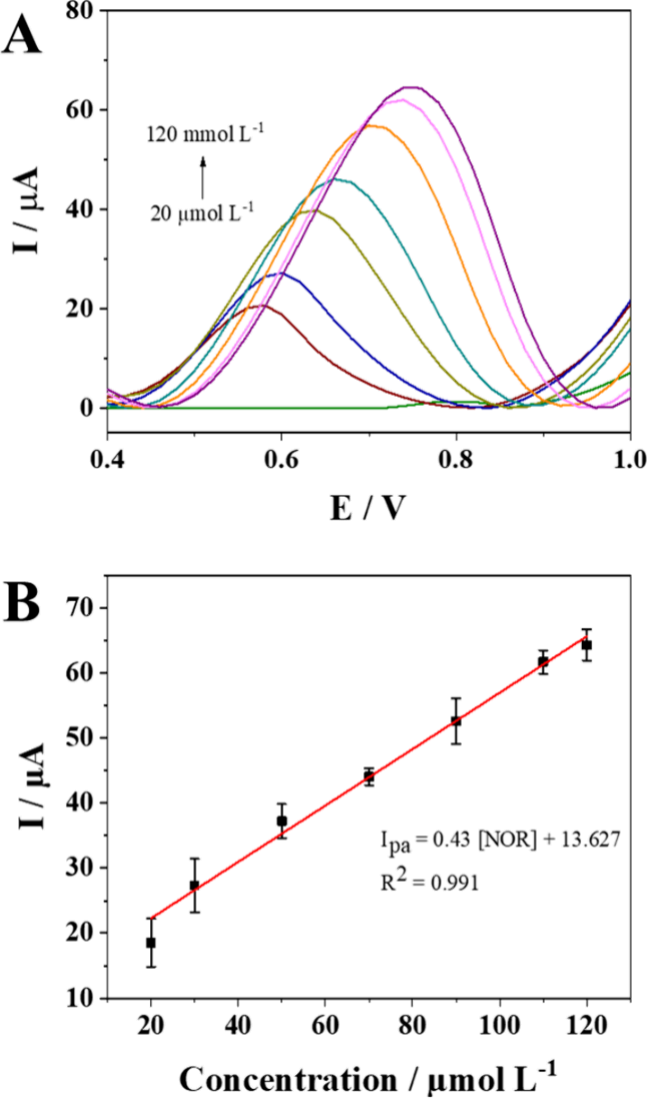
(A) Differential pulse voltammograms obtained after successive additions of NOR to a 0.05 mol L^–1^ Britton–Robinson buffer solution, pH 5.0; (B) Analytical curve of NOR obtained from the voltammograms.

The selectivity of the printable sensor was evaluated through the device’s response to other analytes, aiming to determine its specificity for the target of interest. The results presented in Figure S4 demonstrate a small variation in the peak current in the presence of prednisolone, less than 15%. Similarly, azithromycin caused only a minor change in current intensity. In addition to NOR, lomefloxacin – another representative of the fluoroquinolone class – was used to evaluate the sensor selectivity. The results demonstrated that the sensor can detect lomefloxacin with a sensitivity of 91%. The high sensitivity and selectivity toward fluoroquinolones highlight the sensor’s potential for application in real sample analysis.

### 3.6 Application of the Developed Sensor in Real River Water Samples

The analyses were performed using the standard addition method by DPV, followed by the calculation of recovery percentages. The results demonstrated high recovery rates, ranging from 83.9 to 103.3% (Table 1). This indicates the applicability of the method for NOR quantification in real samples without significant matrix interference.

**Table 1:** Determination of NOR in River Water Samples

added concentration/μmol L^–1^	detected concentration/μmol L^–1^	recovery/%
30	31.5 ± 1.6	103.3
70	70.0 ± 1.5	100.0
110	92.3 ± 7.2	83.9

### 3.7 Comparison with Previous Methods Reported in the Literature

The performance of the sensor proposed in this work was compared with other electrochemical sensors reported in the literature for the quantitative determination of NOR in different matrices. It is noteworthy that there are no studies in the literature referring to electrodes printed with an ink composed of Gr, CB, and SGV for the detection of NOR. It is noted that the selected works (Table 2) employ different types of electrodes modified with materials that present excellent intrinsic characteristics originating from carbon and modifications. There is currently a range of materials to be used in modifications, such as carbonaceous materials, metallic nanoparticles, polymers, metal–organic frameworks, among others.^31,32^ The detection limits obtained for each modified electrode vary, depending on the specific characteristics of the surface modification and the experimental conditions employed.

**Table 2:** Comparison of the Analytical Performance of the Proposed Sensor with Those of Other Electrochemical Sensors for NOR Determination

proposed sensor	technique	linear range/μmol L^–1^	LOD/μmol L^–1^	reference
CF	AMP^a^	1.6–30.0	0.5	^33^
CuO/MWCNTs/GCE	DPV	1.0–47.7	0.3	^34^
MIP/MWCNT/GCE	SWV^b^	0.1–8.0	0.04	^35^
Ni/NiO/C/β-CD/RGO	DPV	0.4–80.0	0.01	^36^
APT-BDD	SWV^b^	0.157–12.5	0.04	^37^
MWCNTCPE/pRGO-ANSA/Au	DPV	0.03–1.0	0.016	^38^
		1.0–50.0		
Au–AgANCCs/fMWCNTsCPE/ChCl	SWV^b^	0.0009–200	0.00014	^39^
Gr-CB-SGV/PET	DPV	20.0–120	0.227	this work

a Amperometry.

b Square Wave Voltammetry.

The use of screen printing to produce the sensor developed in this work, combined with the use of low-cost materials such as conductive ink based on Gr, CB, and SVG, makes the manufacturing process simple and accessible, in addition to providing large-scale production, ensuring reproducibility and good analytical performance. The miniaturized and disposable design of the sensor expands its application potential in various areas.

## 4 Conclusions

The screen-printing technique offered a simple and cost-effective route for large-scale production of printed electrodes with high reproducibility and customization. The conductive ink developed based on Gr, CB, and SGV proved to be easy to prepare and apply, presenting good homogeneity and stability. The structural properties of CB, such as its high surface area and electrical conductivity, in combination with Gr, provided the conductive ink with excellent adhesion to the PET substrate and high conductivity, resulting in a more intense and well-defined voltammetric response. The results obtained demonstrated that the developed sensor showed potential for the determination of NOR in aqueous samples. The method demonstrated good analytical performance, with a LOD of 0.227 μmol L^–1^ and LOQ of 0.756 μmol L^–1^. Selectivity studies indicated that the developed sensor has a high recognition capacity for NOR, as well as specificity in relation to the fluoroquinolone class. The application of the sensor for the determination of NOR in river water samples resulted in good recovery values, between 84 and 103%, demonstrating its efficiency in analytical detection.

## Supplementary Material



## Notes

Additional results regarding the effect of scan rate on the electrochemical response of 5.0 mmol L^–1^ K_4_[Fe­(CN)_6_] in 0.1 mol L^–1^ phosphate buffer (pH 7.0); comparison of the electrochemical responses of Gr and Gr–CB electrodes; Nyquist plots for the different configurations of the printed electrode and cycling stability of the sensor evaluated by cyclic voltammetry; and selectivity study of the printable sensor (PDF)

## References

[1] Demeestere K., Dewulf J., Witte B. D., Langenhove H. V. (2007). Sample
preparation for the analysis of volatile organic compounds in air
and water matrices. J. Chromatogr. A.

[2] Sauvé S., Desrosiers M. (2014). A review of what is an emerging contaminant. Chem. Cent. J..

[3] Nilsen E., Smalling K. L., Ahrens L., Gros M., Miglioranza K. S. B., Picó Y., Heiko L., Schoenfuss H. L. (2019). Critical
review: Grand challenges in assessing the adverse effects of contaminants
of emerging concern on aquatic food webs. Environ.
Toxicol. Chem..

[4] Shawky M., Suleiman W. B., Farrag A. A. (2021). Antibacterial Resistance Pattern
in Clinical and Non-clinical Bacteria by Phenotypic and Genotypic
Assessment. J. Pure Appl. Microbiol..

[5] Mancuso G., Midiri A., Gerace E., Biondo C. (2021). Bacterial Antibiotic
Resistance: The Most Critical Pathogens. Pathogens.

[6] Appelbaum P. C., Hunter P. A. (2000). The fluoroquinolone antibacterials: past, present and
future perspectives. Int. J. Antimicrob. Agents.

[7] Ribeiro T. A. N., de Paula D. D. M., Borges M. M. C., Calixto L. A., Borges K. B. (2025). Sample preparation approaches for determination of
quinolones in aqueous matrixes: systematic review. ACS Meas. Sci. Au.

[8] Dodd-Butera, T. ; Broderick, M. Ciprofloxacin. Encyclopedia of Toxicology; Elsevier, 2014, 966–968.

[9] Larsson D. G. J., Flach C. F. (2022). Antibiotic resistance in the environment. Nat. Rev. Microbiol..

[10] McMullan B. J., Mostaghim M. (2015). Prescribing azithromycin. Aust Prescr..

[11] Stoytcheva M., Velkova Z., Gochev V., Valdez B., Curiel M. (2025). Advances in
electrochemical sensors for paracetamol detection: Electrode materials,
modifications, and analytical applications. Int. J. Electrochem. Sci..

[12] Hong S., Oh S., Kim E., Park E., Chun H. C., Kim I. T., Kim Y. R. (2024). Fabrication of screen-printed electrodes with long-term
stability for voltammetric and potentiometric applications. Sens. Actuators Rep..

[13] Fernandez C., Heger Z., Kizek R., Ramakrishnappa T., Borun A., Faisal N. H. (2015). Pharmaceutical electrochemistry:
the electrochemical oxidation of paracetamol and its voltammetric
sensing in biological samples based on screen printed graphene electrodes. Int. J. Electrochem. Sci..

[14] Fabiani L., Saroglia M., Galatà G., De Santis R., Fillo S., Luca V., Faggioni G., D’Amore N., Regalbuto E., Salvatori P., Terova G., Moscone D., Lista F., Arduini F. (2021). Magnetic beads combined with carbon
black-based screen-printed electrodes for COVID-19: A reliable and
miniaturized electrochemical immunosensor for SARS-CoV-2 detection
in saliva. Biosens Bioelectron..

[15] Camargo J. R., Fernandes-Junior W. S., Azzi D. C., Rocha R. G., Faria L. V., Richter E. M., Muñoz R. A. A., Janegitz B. C. (2022). Development of new
simple compositions of silver inks for the preparation of pseudo-reference
electrodes. Biosensors.

[16] Yao W., Zheng Z., Zhong G., Lin Y., Liu D., Song J., Zhu Y. (2023). Polyethylene terephthalate-based
cathode current collectors coated by ultrathin aluminum metal layers
for commercial lithium-ion batteries with high security and long-term
cycling stability. J. Alloys Compd..

[17] Jurkiewicz K., Pawlyta M., Burian A. (2018). Structure of carbon materials explored
by local transmission electron microscopy and global powder diffraction
probes. C.

[18] Bard, A. J. ; Falkner, L. R. Electrochemical methods fundamentals and applications. 2 ed.; John Wiley & Sons: New York, TX, 2001.

[19] Islam N., Das M., Johan B. A., Shah S. S., Alzahrani A. S., Aziz M. A. (2025). Multifunctional screen-printed conductive inks: design
principles, performance challenges, and application horizons. ACS Appl. Electron. Mater..

[20] Hofland A. (2012). Alkyd resins:
From down and out to alive and kicking. Prog.
Org. Coat..

[21] Lagzdina E., Lingis D., Plukis A., Plukienė R., Germanas D., Garbaras A., Garankin J., Gudelis A., Ignatjev I., Niaura G., Krutovcov S., Remeikis V. (2022). Structural and radiological characterization of irradiated
RBMK-1500 reactor graphite. Nucl. Eng. Technol..

[22] Grulke E. A., Rice S. B., Xiong J. C., Yamamoto K., Yoon T. H., Thomson K., Saffaripour M., Smallwood G. J., Lambert J. W., Stromberg A. J., Macy R., Briot N. J., Qian D. (2018). Size and shape distributions of carbon black aggregates by transmission
electron microscopy. Carbon.

[23] Işeri-Çaǧlar D., Baştürk E., Oktay B., Kahraman M. V. (2014). Preparation
and evaluation of linseed oil based alkyd paints. Prog. Org. Coat..

[24] Elba M. E., Rehim E. M. A., Ashery R. E. (2018). Synthesis and characterization of
alkyd resin based on soybean oil and glycerin using zirconium octoate
as catalyst. Int. J. Chem. Technol..

[25] Pradela-Filho L. A., Andreottic I. A. A., Carvalho J. H. S., Araújo D. A. G., Orzaric L. O., Gattic A., Takeuchi R. M., Santos A. L., Janegitz B. C. (2020). Glass varnish-based carbon conductive ink: A new way
to produce disposable electrochemical sensors. Sens. Actuators B: Chem..

[26] Cândido T. C. O., Pereira A. C., Silva D. N. (2023). Development and characterization
of conductive ink composed of graphite and carbon black for application
in printed electrodes. Analytica.

[27] Oliveira A. E. F., Pereira A. C. (2021). Development of a simple and cheap conductive graphite
ink. J. Electrochem. Soc..

[28] Sui M., Zhou Y., Sheng L., Duan B. (2012). Adsorption of norfloxacin
in aqueous solution by Mg–Al layered double hydroxides with
variable metal composition and interlayer anions. Chem. Eng. J..

[29] Bard, A. J. ; Faulkner, L. R. ; White, H. S. Electrochemical Methods: Fundamentals and Applications. 3rd ed.; Wiley, 2022.

[30] Fix, G. ; Negreli, R. S. ; Coldibeli, B. ; Sartori, E. R. Electrochemical Reaction Mechanism of Antibiotics Explored in Working Electrodes Modified with Nanomaterials. Top. Catal. 2025. 10.1007/s11244-025-02180-2

[31] Kuntoji G., Kousar N., Gaddimath S., Koodlur Sannegowda L. (2024). Macromolecule–Nanoparticle-Based
Hybrid Materials for Biosensor Applications. Biosensors.

[32] C
P K. P., Aralekallu S., Koodlur Sannegowda L. (2024). Efficacy of Phthalocyanine-Based
Catalysts in Electrochemical Sensors: A Comprehensive Review. Adv. Sensor Res..

[33] Morais C. C. O., Magalhães K. F., dos Santos E. V., Castro S. S. L., Martínez-Huitle C. A. (2024). Electrochemically
simple, sensitive, and clean method for monitoring norfloxacin in
advanced oxidative processes. J. Electroanal.
Chem..

[34] Devaraj M., Deivasigamani R. K., Jeyadevan S. (2013). Enhancement of the electrochemical
behavior of CuO nanoleaves on MWCNTs/GC composite film modified electrode
for determination of norfloxacin. Colloids Surf.,
B.

[35] Silva H., Pacheco J., Silva J., Viswanathan S., Delerue-Matos C. (2015). Molecularly imprinted sensor for voltammetric detection
of norfloxacin. Sens. Actuator B: Chem..

[36] Cui X., Cao D., Djellabi R., Qiao M., Wang Y., Zhao S., Mao R., Gong Y., Zhao X., Yang B. (2019). Enhancement of Ni/NiO/graphitized
carbon and β-Cyclodextrin/reduced graphene oxide for the electrochemical
detection of norfloxacin in water sample. J.
Electroanal. Chem..

[37] Karahan F., Basi Z., Keskin E., Pınar P. T., Yardım Y., Sentürk Z. (2020). Electrochemical determination of
fluoroquinolone antibiotic norfloxacin in the presence of anionic
surfactant using the anodically pretreated boron-doped diamond electrode. ChemistrySelect.

[38] Liu Z., Jin M., Cao J., Wang J., Wang X., Zhou G., Van Den Berg A., Shui L. (2018). High sensitive electrochemical sensor
for determination of norfloxacin and its metabolism using MWCNT-CPE/pRGO-ANSA/Au. Sens. Actuators B Chem..

[39] Adane W. D., Chandravanshi B. S., Getachew N., Tessema M. (2024). A cutting-edge electrochemical
sensing platform for the simultaneous determination of the residues
of antimicrobial drugs, rifampicin and norfloxacin, in water samples. Anal. Chim. Acta.

