# Supplementary material for: Printable Carbon Black/Graphite Conductive Ink toward Electrochemical Determination of Norfloxacin in Environmental Samples
Source: ACS Omega. 2025 Oct 10;10(41):49207–15. doi: 10.1021/acsomega.5c08667 (PMC12573298; doi:10.1021/acsomega.5c08667)
Supplement: Supplementary file 1 [file ao5c08667_si_001.pdf]

Supplementary material

Printable carbon black/graphite conductive ink toward electrochemical determination of norfloxacin in environmental samples

Marcella Matos Cordeiro Borges*, Thaís Cristina de Oliveira Cândido, Arnaldo César Pereira*

Departamento de Ciências Naturais, Universidade Federal de São João del-Rei (UFSJ), Campus Dom Bosco, Praça Dom Helvécio 74, Fábricas, 36301-160, São João del-Rei, Minas Gerais, Brazil

Correspondence:

Dr. Marcella Matos Cordeiro Borges, Departamento de Ciências Naturais, Universidade Federal de São João del-Rei, Campus Dom Bosco, Praça Dom Helvécio 74, Fábricas, 36301-160, São João del-Rei, Minas Gerais, Brazil.

e-mail: marcellamatosc@hotmail.com; phone number: +55 32 3379 – 5163

Prof. Arnaldo César Pereira, Departamento de Ciências Naturais, Universidade Federal de São João del-Rei, Campus Dom Bosco, Praça Dom Helvécio 74, Fábricas, 36301-160, São João del-Rei, Minas Gerais, Brazil.

e-mail: arnaldo@ufsj.edu.br; phone number: +55 32 3379 – 5163

Figures

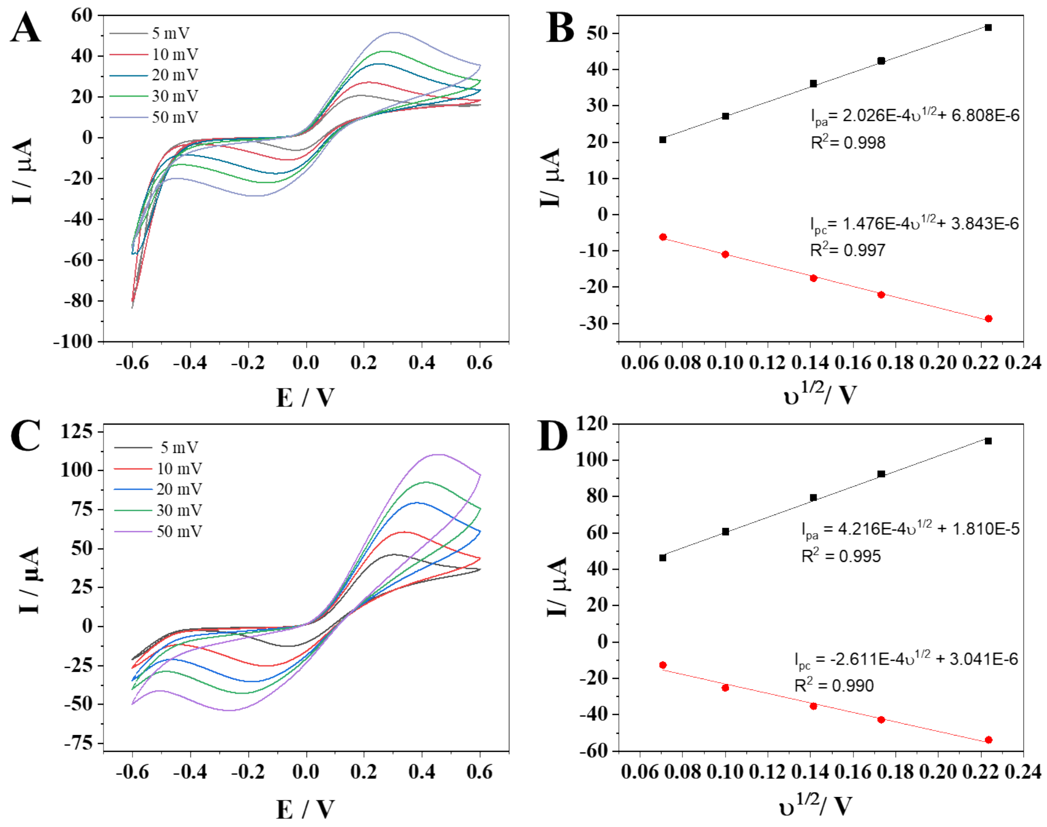

Figure S1. Effect of scan rate on the electrochemical response of 5.0 mmol L^{-1} $K_4[Fe(CN)_6]$ in 0.1 mol L^{-1} phosphate buffer (pH 7.0). (A) Cyclic voltammograms obtained for the printed electrode using Gr and SGV ink at different scan rates (5, 10, 20, 30, 50, and 100 mV s^{-1}); (B) Plot of peak current versus the square root of the scan rate corresponding to the voltammograms in (A); (C) Cyclic voltammograms obtained for the printed electrode using Gr, CB, and SGV ink at different scan rates (5, 10, 20, 30, 50, and 100 mV s^{-1}); (D) Plot of peak current versus the square root of the scan rate corresponding to the voltammograms in (C).

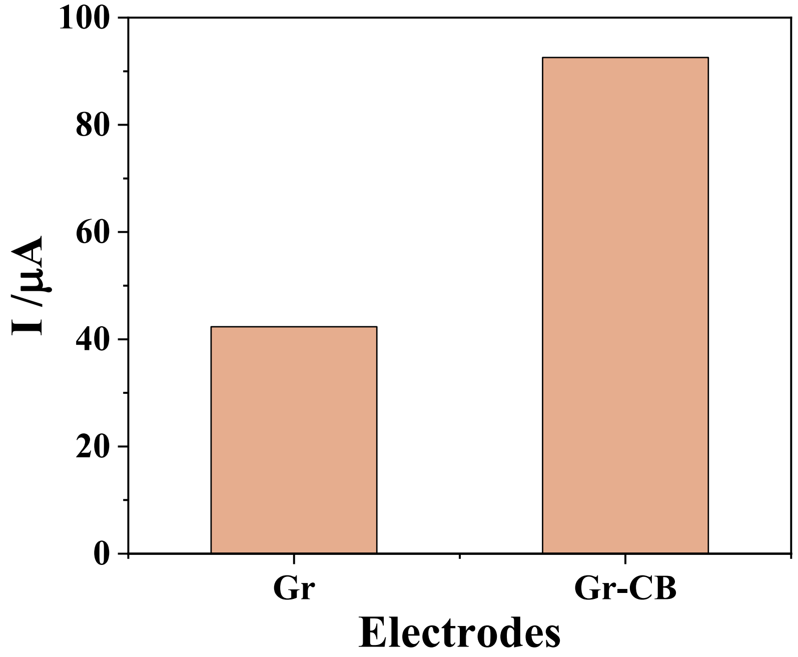

Figure S2. Comparison of the electrochemical responses of Gr and Gr–CB electrodes.

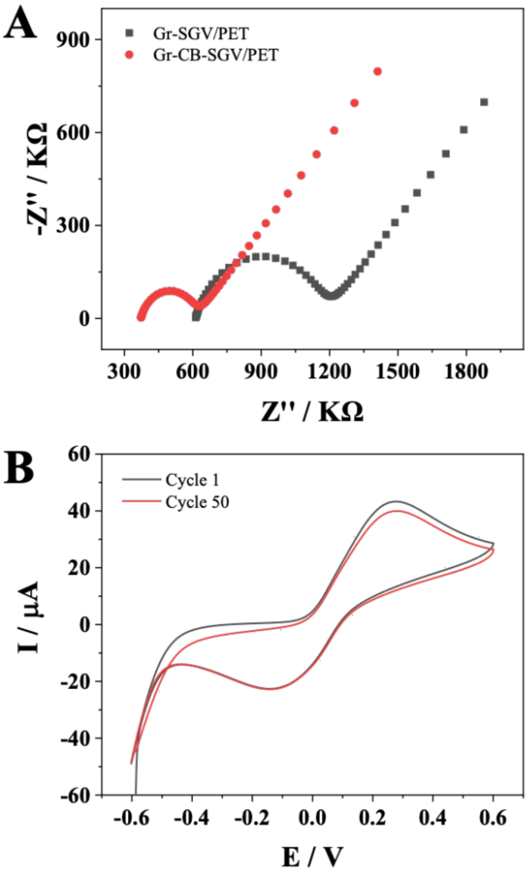

Figure S3. (A) Nyquist plots for the different configurations of the printed electrode using 5.0 mmol L^{-1} $K_3[Fe(CN)_6]/K_4[Fe(CN)_6]$ in 0.1 mol L^{-1} KCl at pH 7.0.; (B) Cycling stability of the sensor evaluated by cyclic voltammetry at a scan rate of 30 mV s^{-1} in 0.1 mol L^{-1} phosphate buffer at pH 7.0 containing 5.0 mmol L^{-1} $K_4[Fe(CN)_6]$.

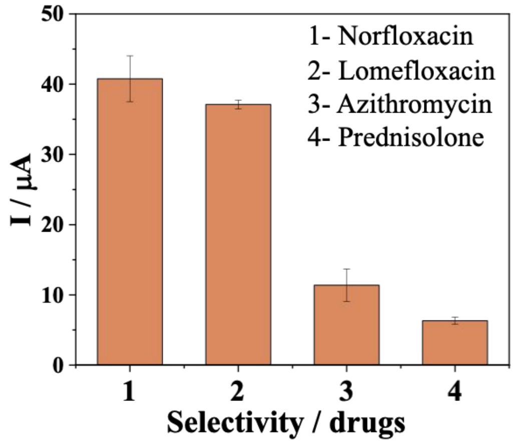

Figure S4. Selectivity study of the printable sensor.
